# An Inertial Proximal-Gradient Penalization Scheme for Constrained Convex Optimization Problems

**DOI:** 10.1007/s10013-017-0256-9

**Published:** 2017-09-01

**Authors:** Radu Ioan Boţ, Ernö Robert Csetnek, Nimit Nimana

**Affiliations:** 1 Faculty of Mathematics, University of Vienna, Oskar-Morgenstern-Platz 1, 1090 Vienna, Austria; 2 Department of Mathematics, Faculty of Science, Naresuan University, Phitsanulok, 65000 Thailand

**Keywords:** Proximal-gradient algorithm, Inertial algorithm, Penalization, Fenchel conjugate, 47H05, 65K05, 90C25

## Abstract

We propose a proximal-gradient algorithm with penalization terms and inertial and memory effects for minimizing the sum of a proper, convex, and lower semicontinuous and a convex differentiable function subject to the set of minimizers of another convex differentiable function. We show that, under suitable choices for the step sizes and the penalization parameters, the generated iterates weakly converge to an optimal solution of the addressed bilevel optimization problem, while the objective function values converge to its optimal objective value.

## Introduction and Preliminaries

Let ${\mathcal H}$ be a real Hilbert space with inner product 〈⋅,⋅〉 and associated norm ∥⋅∥. Let $f:{\mathcal H}\rightarrow \overline {\mathbb R}=\mathbb {R}\cup \{\pm \infty \}$ be a proper, convex, and lower semicontinuous function, and $h:{\mathcal H}\to \mathbb {R}$ and $g:{\mathcal H}\to \mathbb {R}$ be convex and differentiable functions with Lipschitz continuous gradients with positive Lipschitz constants *L*_*h*_ and *L*_*g*_, respectively. We consider the bilevel optimization problem

1 $$ \min_{x\in\arg\min g} f(x)+h(x) $$

and assume that the set

$$\mathcal{S}:=\arg\min\{f(x)+h(x):x\in\arg\min g\} $$

is nonempty. We also assume without loss of generality that min*g* = 0.

The work of Attouch and Czarnecki [5] has represented the starting point of a series of articles approaching the minimization of a smooth or (sometimes) a complexly structured nonsmooth objective function subject to the set of minimzers of another function, from either a discrete perspective through iterative numerical algorithms or a continuous one through dynamical systems (see [4–8, 11, 17–19, 24–27, 30, 37, 39]). The function determining the feasible set is evaluated in both settings in the spirit of penalty methods and contributes to the convergence of the generated sequences, in the discrete setting, and to the asymptotic convergence of the generated trajectories, in the continuous setting, to an optimal solution of the underlying bilevel optimization problem. We emphasize in particular the proximal-gradient algorithm with penalty term which has been introduced in [8] and for which weak ergodic convergence has been proved.

In this paper, we consider this algorithm in the context of solving problem (1) and we enhance it with inertial and memory effects. Our aim is to provide suitable choices for the step sizes and the penalization parameters, such that the generated iterates weakly converge to an optimal solution of the problem (1), while the objective function values converge to its optimal objective value. Algorithms of inertial type follow by time discretization of differential inclusions of second-order type (see [1, 3]) and have been first investigated in the context of the minimization of a differentiable function by Polyak in [40] and Bertsekas in [14]. In the last two decades, intensive research efforts dedicated to algorithms of inertial type and to their convergence behavior can be noticed (see [1–3, 10, 20–23, 28, 29, 31–36, 38]). For a variety of situations, in particular in the context of solving real-world problems, the presence of inertial terms improves the convergence behavior of the generated sequences. It is also well-known (see [9, 13]) that enhancing the proximal-gradient algorithm with inertial effects may lead to a considerable improvement of the convergence behavior of the sequence of objective function values.

The proximal-gradient algorithm with penalization terms and inertial and memory effects we propose for solving (1) is the following.

### **Algorithm 1**

Initialization: Choose positive sequences $\{\lambda _{n}\}_{n=1}^{\infty }$, $\{\beta _{n}\}_{n=1}^{\infty }$, and a nonnegative constant *α* ∈ [0, 1). Take arbitrary $x_{0},x_{1}\in {\mathcal H}$. Iterative step: For every *n* ≥ 1 and given current iterates $x_{n-1}, x_{n}\in {\mathcal H}$ define $x_{n+1} \in {\mathcal H}$ by

$$x_{n+1}:=\text{prox}_{\lambda_{n}f}\left( x_{n}+\alpha(x_{n}-x_{n-1})-\lambda_{n}\nabla h(x_{n})-\lambda_{n}\beta_{n}\nabla g(x_{n})\right). $$

For $x \in {\mathcal H}$ we denote by $\text {prox}_{\lambda _{n} f}(x)$ the proximal point of the function *f* of parameter *λ*_*n*_ at *x*, which is the unique optimal solution of the optimization problem

$$ \inf_{y\in {\mathcal H}}\left\{f(y)+\frac{1}{2\lambda_{n}}\|y-x\|^{2}\right\}. $$

In Algorithm 1, $\{\lambda _{n}\}_{n=1}^{\infty }$ denotes the sequence of step sizes, $\{\beta _{n}\}_{n=1}^{\infty }$ the sequence of penalization parameters, and *α* ∈ [0, 1) the parameter that controls the inertial terms.

The proposed numerical scheme recovers, when *α* = 0, the algorithm investigated in [37] and, under the additional assumption *f* = 0, the gradient method of penalty type from [39]. In the case *f* = 0, Algorithm 1 gives rise to the gradient method of penalty type with inertial and memory effects introduced and studied in [30].

We prove weak convergence for the generated iterates to an optimal solution of (1), by making use of generalized Fejér monotonicity techniques and of the Opial lemma. The performed analysis allows us also to show the convergence of the objective function values to the optimal objective value of (1).

In the remaining of this section we recall some elements of convex analysis. For a function $f:{\mathcal H}\rightarrow \overline {\mathbb {R}}$ we denote by $\text {dom}\,f=\{x\in {\mathcal H}:f(x)<+\infty \}$ its effective domain and say that *f* is proper, if dom *f*≠*∅* and *f*(*x*)≠ −*∞* for all $x\in {\mathcal H}$. Let $f^{\ast }:{\mathcal H} \rightarrow \overline {\mathbb R}$, $f^{\ast }(u)=\sup _{x\in {\mathcal H}}\{\langle u,x\rangle -f(x)\}$ for all $u\in {\mathcal H}$, be the conjugate function of *f*. The subdifferential of *f* at $x\in {\mathcal H}$, with $f(x)\in \mathbb {R}$, is the set $\partial f(x):=\{v\in {\mathcal H}:f(y)\geq f(x)+\langle v,y-x\rangle ~ \forall y\in {\mathcal H}\}$. We take by convention *∂* *f*(*x*) := *∅*, if *f*(*x*) ∈{±*∞*}. We also denote by $\min f := \inf _{x \in {\mathcal H}} f(x)$ the optimal objective value of the function *f* and by $\arg \min f :=\{x \in {\mathcal H}: f(x) = \min f\}$ its set of global minima.

A convex and differentiable function $g:{\mathcal H}\to \mathbb {R}$ has a Lipschitz continuous gradient with Lipschitz constant *L*_*g*_ > 0, if ∥∇*g*(*x*) −∇*g*(*y*)∥≤ *L*_*g*_∥*x* − *y*∥ for all $x,y \in {\mathcal H}$. It is well-known (see, for instance, [12, Theorem 18.15]) that this is equivalent to ∇*g* is $\frac {1}{L_{g}}$-cocoercive, namely, $\langle x-y, \nabla g(x) - \nabla g(y) \rangle \geq \frac {1}{L_{g}} \|\nabla g(x) - \nabla g(y)\|^{2}$ for all $x,y \in {\mathcal H}$.

Let $M\subseteq {\mathcal H}$ be a nonempty set. The indicator function of *M*, $\delta _{M}:{\mathcal H}\rightarrow \overline {\mathbb {R}}$, is the function which takes the value 0 on *M* and + *∞* otherwise. The subdifferential of the indicator function is the normal cone of *M*, that is $N_{M}(x)=\{u\in {\mathcal H}:\langle u,y-x\rangle \leq 0~ \forall y\in M\}$, if *x* ∈ *M* and *N*_*M*_(*x*) = *∅* for *x*∉*M*. For *x* ∈ *M*, we have that *u* ∈ *N*_*M*_(*x*) if and only if *σ*_*M*_(*u*) = 〈*u*,*x*〉, where *σ*_*M*_ is the support function of *M*, defined by $\sigma _{M} : {\mathcal H} \rightarrow \overline {\mathbb {R}}, \sigma _{M}(u)=\sup _{y\in M}\langle y,u\rangle $. Finally, ran(*N*_*M*_) denotes the range of the normal cone *N*_*M*_, that is, *p* ∈ran(*N*_*M*_) if and only if there exists *x* ∈ *M*, such that *p* ∈ *N*_*M*_(*x*).

## Technical Lemmas

The setting in which we will carry out the convergence analysis for Algorithm 1 is settled by the following hypotheses.

### **Assumption 1**

(I) The subdifferential sum formula *∂*(*f* + *δ*_arg min *g*_) = *∂* *f* + *N*_arg min *g*_ holds;
(II) The objective function *f* + *h* is bounded from below;
(III) There exist positive constants *η*_0_,*a*,*b*,*K* and *c* > 1, such that for every *n* ≥ 1:
  $$0<a\leq \lambda_{n}\beta_{n}\leq b<\frac{2}{L_{g}(1+\eta_{0})^{2}}, \frac{L_{h}+\beta_{n}L_{g}}{2}+\frac{\alpha-1}{\lambda_{n}}\leq -(1+2\alpha)K-c $$
  and
  $$\beta_{n+1}-\beta_{n}\leq K\frac{\eta_{0}}{1+\eta_{0}}\lambda_{n+1}\beta_{n+1}; $$
(IV) $\{\lambda _{n}\}_{n=1}^{\infty }\in \ell ^{2}\setminus \ell ^{1}$ and $\left (\frac {1}{\lambda _{n+1}}-\frac {1}{\lambda _{n}}\right )\alpha \leq 2$ for every *n* ≥ 1;
(V) ${\sum }_{n=1}^{\infty }\lambda _{n}\beta _{n}\left [g^{\ast }\left (\frac {p}{\beta _{n}}\right )-\sigma _{\arg \min g}\left (\frac {p}{\beta _{n}}\right )\right ]\!\!<\!+\infty $ for every *p* ∈ran(*N*_arg min *g*_).

### *Remark 1*

For conditions which guarantee exact convex subdifferential sum formulas, we refer to [12, 15, 16, 41]. One of these conditions, which is frequently fulfilled in applications, asks for the continuity of the function f and thus does not require any knowledge of the set of minimizers of g.

The assumption in (V) originates from the work of Attouch and Czarnecki [5]; we refer to [4–8, 11, 17–19, 24–27, 30, 37, 39] for other variants, generalizations to monotone operators, and concrete examples for which this condition is satisfied (see also Remark 2).

The aim of the following three results is to derive a generalized Fejér-type inequality in the spirit of the one in the hypotheses of Lemma 4. This will be achieved in terms of the sequence (Γ_*n*_)_*n*≥1_, defined before Lemma 3, and which can be seen as a Lyapunov sequence equal to the sum of the objective function and a penalization of the function *g* both at the current iterate, and the distance of the current iterate to a fixed optimal solution.

### **Lemma 1**

*Let* $u\in \mathcal {S}$. *According to the first-order optimality conditions, there exist* *v* ∈ *∂* *f*(*u*)*and* *p* ∈ *N*_arg min *g*_(*u*), *such that* 0 = *v* + ∇*h*(*u*) + *p*. *Set* *φ*_*n*_ := ∥*x*_*n*_ − *u*∥^2^ *for every* *n* ≥ 1. *Then, for every* *n* ≥ 1*and* *η* > 0, *it holds that*

2 $$\begin{array}{@{}rcl@{}} \varphi_{n+1}&-&\varphi_{n}-\alpha(\varphi_{n}-\varphi_{n-1}) +\lambda_{n}\beta_{n}\left[\frac{2}{L_{g}(1+\eta)}-(1+\eta)\lambda_{n}\beta_{n}\right]\|\nabla g(x_{n})\|^{2}\\ &+&\frac{\eta}{1+\eta}\lambda_{n}\beta_{n}g(x_{n})\leq 2\alpha\|x_{n}-x_{n-1}\|^{2}+\|x_{n+1}-x_{n}\|^{2}\\ &&+\frac{\,4(1+\eta)}{\eta}{\lambda_{n}^{2}}\|\nabla h(u)+v\|^{2}\\ &&+\lambda_{n}\left[\frac{4(1+\eta)}{\eta}\lambda_{n}-\frac{2}{L_{h}}\right]\|\nabla h(x_{n})-\nabla h(u)\|^{2}\\ &&+\frac{\eta}{1+\eta}\lambda_{n}\beta_{n}\left[g^{\ast}\left( \frac{2p}{\frac{\eta}{1+\eta}\beta_{n}}\right)-\sigma_{\arg\min g}\left( \frac{2p}{\frac{\eta}{1+\eta}\beta_{n}}\right)\right]. \end{array} $$

### *Proof*

Set *y*_*n*_ := *x*_*n*_ + *α*(*x*_*n*_ − *x*_*n*−1_) for every *n* ≥ 1. Since *y*_*n*_ − *x*_*n*+1_ − *λ*_*n*_∇*h*(*x*_*n*_) − *λ*_*n*_ *β*_*n*_∇*g*(*x*_*n*_) ∈ *λ*_*n*_ *∂* *f*(*x*_*n*+1_) and *v* ∈ *∂* *f*(*u*), the monotonicity of *∂* *f* guarantees that

$$\langle y_{n}-x_{n+1}-\lambda_{n}\nabla h(x_{n})-\lambda_{n}\beta_{n}\nabla g(x_{n})-\lambda_{n}v,x_{n+1}-u\rangle\geq 0 \quad \forall n \geq 1 $$

or, equivalently,

3 $$ 2\langle y_{n}-x_{n+1},u-x_{n+1}\rangle\leq2\lambda_{n}\langle u-x_{n+1},\nabla h(x_{n})+\beta_{n}\nabla g(x_{n})+v\rangle \quad \forall n\geq1. $$

We notice that for every *n* ≥ 0

4 $$ 2\langle x_{n}-x_{n+1},u-x_{n+1} \rangle = \varphi_{n+1}-\varphi_{n}+\|x_{n+1}-x_{n}\|^{2} $$

and so for every *n* ≥ 1

5 $$\begin{array}{@{}rcl@{}} 2\alpha\langle x_{n}-x_{n-1},u-x_{n+1} \rangle &=& 2\alpha\langle x_{n}-x_{n-1},u-x_{n} \rangle + 2\alpha\langle x_{n}-x_{n-1},x_{n}-x_{n+1} \rangle\\ &\geq&\alpha(\|x_{n-1}-u\|^{2}-\|x_{n}-u\|^{2}-\|x_{n}-x_{n-1}\|^{2})\\ &&+\,\alpha(-\|x_{n}-x_{n-1}\|^{2}-\|x_{n+1}-x_{n}\|^{2})\\ &=&\alpha(\varphi_{n-1}-\varphi_{n})-2\alpha\|x_{n}-x_{n-1}\|^{2}\\ &&-\,\alpha\|x_{n+1}-x_{n}\|^{2}. \end{array} $$

By employing (4) and (5) in inequality (3), we obtain for every *n* ≥ 1

6 $$\begin{array}{@{}rcl@{}} \varphi_{n+1}-\varphi_{n} &-&\alpha(\varphi_{n}-\varphi_{n-1})+(1-\alpha)\|x_{n+1}-x_{n}\|^{2}-2\alpha\|x_{n}-x_{n-1}\|^{2}\\ &\leq& 2\lambda_{n}\langle u-x_{n+1},\nabla h(x_{n})+\beta_{n}\nabla g(x_{n})+v\rangle\\ &=& 2\lambda_{n}\langle u-x_{n+1},\beta_{n}\nabla g(x_{n})\rangle+2\lambda_{n}\langle u-x_{n+1},\nabla h(x_{n})+v\rangle\\ &=& 2\lambda_{n}\langle u-x_{n},\beta_{n}\nabla g(x_{n})\rangle+2\lambda_{n}\langle x_{n}-x_{n+1},\beta_{n}\nabla g(x_{n})\rangle\\ &&+\,2\lambda_{n}\langle u-x_{n+1},\nabla h(x_{n})+v\rangle. \end{array} $$

Next, we evaluate the first two terms on the right-hand side in the above statement. Since ∇*g* is $\frac {1}{L_{g}}$-cocoercive, we have

$$\langle\nabla g(x_{n})-\nabla g(u),x_{n}-u\rangle\geq\frac{1}{L_{g}}\|\nabla g(x_{n})-\nabla g(u)\|^{2} \quad \forall n \geq 1, $$

and from here, since ∇*g*(*u*) = 0,

7 $$ \langle\nabla g(x_{n}),u-x_{n}\rangle\leq -\frac{1}{L_{g}}\|\nabla g(x_{n})\|^{2} \quad \forall n\geq1. $$

On the other hand, since *g* is convex and differentiable, we have

$$0=g(u)\geq g(x_{n})+\langle\nabla g(x_{n}),u-x_{n}\rangle \quad \forall n \geq 1 $$

or, equivalently,

8 $$ \langle\nabla g(x_{n}),u-x_{n}\rangle\leq -g(x_{n}) \quad \forall n\geq1. $$

From (7) and (8) we obtain for all *n* ≥ 1

9 $$ 2\lambda_{n}\beta_{n}\langle\nabla g(x_{n}),u-x_{n}\rangle\leq -\frac{2}{L_{g}(1+\eta)}\lambda_{n}\beta_{n}\|\nabla g(x_{n})\|^{2} - \frac{2\eta}{1+\eta}\lambda_{n}\beta_{n}g(x_{n}). $$

For the term 2*λ*_*n*_ *β*_*n*_〈*x*_*n*_ − *x*_*n*+1_,∇*g*(*x*_*n*_)〉 in (6) we have for all *n* ≥ 1 the following estimate

10 $$ 2\lambda_{n}\beta_{n}\langle x_{n}-x_{n+1},\nabla g(x_{n})\rangle\leq \frac{1}{1+\eta}\|x_{n+1}-x_{n}\|^{2}+(1+\eta){\lambda_{n}^{2}}{\beta_{n}^{2}}\|\nabla g(x_{n})\|^{2}. $$

Employing the inequalities (9) and (10) in (6), we obtain for every *n* ≥ 1 that

$$\begin{array}{@{}rcl@{}} \varphi_{n+1}-\varphi_{n} &-&\alpha(\varphi_{n}-\varphi_{n-1})+(1-\alpha)\|x_{n+1}-x_{n}\|^{2}-2\alpha\|x_{n}-x_{n-1}\|^{2}\\ &\leq& -\,\frac{2}{L_{g}(1+\eta)}\lambda_{n}\beta_{n}\|\nabla g(x_{n})\|^{2}-\frac{2\eta}{1+\eta}\lambda_{n}\beta_{n}g(x_{n})\\ &&+\,\frac{1}{1+\eta}\|x_{n+1}-x_{n}\|^{2}+(1+\eta){\lambda_{n}^{2}}{\beta_{n}^{2}}\|\nabla g(x_{n})\|^{2}\\ &&+\,2\lambda_{n}\langle u-x_{n+1},\nabla h(x_{n})+v\rangle, \end{array} $$

and further

11 $$\begin{array}{@{}rcl@{}} \varphi_{n+1}-\varphi_{n} &-& \alpha(\varphi_{n}-\varphi_{n-1})+\left[\frac{2}{L_{g}(1+\eta)}-(1+\eta)\lambda_{n}\beta_{n}\right]\lambda_{n}\beta_{n}\|\nabla g(x_{n})\|^{2}\\ &\leq& 2\alpha\|x_{n}-x_{n-1}\|^{2}-\frac{2\eta}{1+\eta}\lambda_{n}\beta_{n}g(x_{n})\\ &&+\,\left[\frac{1}{1+\eta}+\alpha-1\right]\|x_{n+1}-x_{n}\|^{2}\\ &&+\,2\lambda_{n}\langle u-x_{n+1},\nabla h(x_{n})+v\rangle. \end{array} $$

Not least,

$$\begin{array}{@{}rcl@{}} 2\lambda_{n}\langle u-x_{n+1},\nabla h(x_{n})+v\rangle &=& 2\lambda_{n}\langle u-x_{n},\nabla h(x_{n})+v\rangle\\ &&+\,2\lambda_{n}\langle x_{n}-x_{n+1},\nabla h(x_{n})+v\rangle\\ &\leq& 2\lambda_{n}\langle u-x_{n},\nabla h(x_{n})+v\rangle\\ && +\,\frac{\eta}{2(1+\eta)}\|x_{n+1}-x_{n}\|^{2}\\ &&+\,\frac{2(1+\eta)}{\eta}{\lambda_{n}^{2}}\|\nabla h(x_{n})+v\|^{2}\\ &\leq& 2\lambda_{n}\langle u-x_{n},\nabla h(x_{n})+v\rangle\\ && +\,\frac{\eta}{2(1+\eta)}\|x_{n+1}-x_{n}\|^{2}\\ &&+\,\frac{4(1+\eta)}{\eta}{\lambda_{n}^{2}}\|\nabla h(x_{n})-\nabla h(u)\|^{2}\\ &&+\,\frac{4(1+\eta)}{\eta}{\lambda_{n}^{2}}\|\nabla h(u)+v\|^{2} \end{array} $$

and by employing this estimate in (11), we deduce that for every *n* ≥ 1

12 $$\begin{array}{@{}rcl@{}} \varphi_{n+1}&-&\varphi_{n}-\alpha(\varphi_{n}-\varphi_{n-1}) + \lambda_{n}\beta_{n}\left[\frac{2}{L_{g}(1+\eta)}-(1+\eta)\lambda_{n}\beta_{n}\right]\|\nabla g(x_{n})\|^{2}\\ &+&\frac{\eta}{1+\eta}\lambda_{n}\beta_{n}g(x_{n})\leq 2\alpha\|x_{n}-x_{n-1}\|^{2} \\ &&+\left[\frac{1}{1+\eta}+\frac{\eta}{2(1+\eta)}+\alpha-1\right]\|x_{n+1}-x_{n}\|^{2}\\ &&+\frac{4(1+\eta)}{\eta}{\lambda_{n}^{2}}\|\nabla h(x_{n})-\nabla h(u)\|^{2}\\ &&+\frac{4(1+\eta)}{\eta}{\lambda_{n}^{2}}\|\nabla h(u)+v\|^{2}\\ &&-\frac{\eta}{1+\eta}\lambda_{n}\beta_{n}g(x_{n})+2\lambda_{n}\langle u-x_{n},\nabla h(x_{n})+v\rangle. \end{array} $$

By using the $\frac {1}{L_{h}}$-cocoercivity of ∇*h* we obtain for every *n* ≥ 1 that

$$\begin{array}{@{}rcl@{}} &&2\lambda_{n}\langle u-x_{n},\nabla h(x_{n})+v\rangle-\frac{\eta}{1+\eta}\lambda_{n}\beta_{n}g(x_{n})\\ &=& 2\lambda_{n}\langle u-x_{n},\nabla h(x_{n})-\nabla h(u)\rangle+2\lambda_{n}\langle u-x_{n},\nabla h(u)+v\rangle-\frac{\eta}{1+\eta}\lambda_{n}\beta_{n}g(x_{n})\\ &\leq& \frac{-2\lambda_{n}}{L_{h}}\|\nabla h(x_{n})-\nabla h(u)\|^{2}+2\lambda_{n}\langle u-x_{n},-p\rangle-\frac{\eta}{1+\eta}\lambda_{n}\beta_{n}g(x_{n}), \end{array} $$

while, since *p* ∈ *N*_arg min *g*_(*u*), it holds that

$$\begin{array}{@{}rcl@{}} &&-\,2\lambda_{n}\langle u-x_{n},p\rangle-\frac{\eta}{1+\eta}\lambda_{n}\beta_{n} g(x_{n})\\ &=& 2\lambda_{n}\langle x_{n},p\rangle-\frac{\eta}{1+\eta}\lambda_{n}\beta_{n} g(x_{n})-2\lambda_{n}\langle u, p \rangle\\ &=&\frac{\eta}{1+\eta}\lambda_{n}\beta_{n}\left[\left\langle x_{n},\frac{2p}{\frac{\eta}{1+\eta}\beta_{n}}\right\rangle-g(x_{n})-\left\langle u,\frac{2p}{\frac{\eta}{1+\eta}\beta_{n}}\right\rangle\right]\\ &\leq&\frac{\eta}{1+\eta}\lambda_{n}\beta_{n}\left[g^{*}\left( \frac{2p}{\frac{\eta}{1+\eta}\beta_{n}}\right)-\left\langle u,\frac{2p}{\frac{\eta}{1+\eta}\beta_{n}}\right\rangle\right]\\ &=&\frac{\eta}{1+\eta}\lambda_{n}\beta_{n}\left[g^{*}\left( \frac{2p}{\frac{\eta}{1+\eta}\beta_{n}}\right)-\sigma_{\arg\min g}\left( \frac{2p}{\frac{\eta}{1+\eta}\beta_{n}}\right)\right] \quad \forall n \geq 1. \end{array} $$

By combining these two inequalities with (12), it follows for every *n* ≥ 1 that

$$\begin{array}{@{}rcl@{}} \varphi_{n+1}&-&\varphi_{n}-\alpha(\varphi_{n}-\varphi_{n-1}) +\lambda_{n}\beta_{n}\left[\frac{2}{L_{g}(1+\eta)}-(1+\eta)\lambda_{n}\beta_{n}\right]\|\nabla g(x_{n})\|^{2}\\ &+&\frac{\eta}{1+\eta}\lambda_{n}\beta_{n}g(x_{n})\leq 2\alpha\|x_{n}-x_{n-1}\|^{2}\\ &&+\left[\frac{1}{1+\eta}+\frac{\eta}{2(1+\eta)}+\alpha-1\right]\|x_{n+1}-x_{n}\|^{2}\\ &&+\frac{4(1+\eta)}{\eta}{\lambda_{n}^{2}}\|\nabla h(u)+v\|^{2}\\ &&+\lambda_{n}\left[\frac{4(1+\eta)}{\eta}\lambda_{n}-\frac{2}{L_{h}}\right]\|\nabla h(x_{n})-\nabla h(u)\|^{2}\\ &&+\frac{\eta}{1+\eta}\lambda_{n}\beta_{n}\left[g^{\ast}\left( \frac{2p}{\frac{\eta}{1+\eta}\beta_{n}}\right)-\sigma_{\arg\min g}\left( \frac{2p}{\frac{\eta}{1+\eta}\beta_{n}}\right)\right]. \end{array} $$

Since *α* ∈ [0, 1) and *η* > 0, it holds that $\frac {1}{1+\eta }+\frac {\eta }{2(1+\eta )}+\alpha -1<1$, which together with the inequality above lead to the conclusion. □

For simplicity, we will make use of the following notation:

$${\Omega}_{n}(x_{n}):=f(x_{n})+h(x_{n})+\beta_{n} g(x_{n}) \quad \forall n\geq1. $$

### **Lemma 2**

*For every* *n* ≥ 1*it holds that*

13 $$\begin{array}{@{}rcl@{}} {\Omega}_{n+1}(x_{n+1})-{\Omega}_{n}(x_{n}) &\leq& \left[\frac{\alpha}{2\lambda_{n}}-\frac{1}{\lambda_{n}}+\frac{L_{h}+\beta_{n} L_{g}}{2}\right]\|x_{n+1}-x_{n}\|^{2}\\ &&+\,\frac{\alpha}{2\lambda_{n}}\|x_{n}-x_{n-1}\|^{2}+(\beta_{n+1}-\beta_{n})g(x_{n+1}). \end{array} $$

### *Proof*

Recall that for every *n* ≥ 1 we have $\frac {y_{n}-x_{n+1}}{\lambda _{n}}-\nabla h(x_{n})-\beta _{n}g(x_{n})\in \partial f(x_{n+1})$, which implies

$$f(x_{n})\geq f(x_{n+1})+\left\langle \frac{y_{n}-x_{n+1}}{\lambda_{n}}-\nabla h(x_{n})-\beta_{n}g(x_{n}),x_{n}-x_{n+1} \right\rangle. $$

From here, it follows that for every *n* ≥ 1 we have

14 $$\begin{array}{@{}rcl@{}} f(x_{n+1})-f(x_{n}) &\leq&-\frac{1}{\lambda_{n}}\|x_{n+1}-x_{n}\|^{2}+\frac{\alpha}{\lambda_{n}}\langle x_{n}-x_{n-1},x_{n+1}-x_{n}\rangle\\ &&+\,\langle \nabla h(x_{n}),x_{n}-x_{n+1}\rangle+\beta_{n}\langle \nabla g(x_{n}),x_{n}-x_{n+1}\rangle\\ &\leq& \left[\frac{\alpha}{2\lambda_{n}}-\frac{1}{\lambda_{n}}\right]\|x_{n+1}-x_{n}\|^{2}+\frac{\alpha}{2\lambda_{n}}\|x_{n}-x_{n-1}\|^{2} \\ &&+\,\langle \nabla h(x_{n}),x_{n}-x_{n+1}\rangle+\beta_{n}\langle \nabla g(x_{n}),x_{n}-x_{n+1}\rangle. \end{array} $$

From the Descent Lemma (see for example [12, Theorem 18.15]), we obtain for every *n* ≥ 1 that

$$g(x_{n+1})\leq g(x_{n})+\langle \nabla g(x_{n}), x_{n+1}-x_{n}\rangle+\frac{L_{g}}{2}\|x_{n+1}-x_{n}\|^{2}, $$

and

$$h(x_{n+1})\leq h(x_{n})+\langle \nabla h(x_{n}), x_{n+1}-x_{n}\rangle+\frac{L_{h}}{2}\|x_{n+1}-x_{n}\|^{2}. $$

By combining these relations with inequality (14), we finally obtain the inequality in the statement of the lemma. □

For the forthcoming statements, we fix an element $u\in \mathcal {S}$. For a simpler formulation of these results, we will use the following notation:

$$\begin{array}{@{}rcl@{}} {\Gamma}_{n}&:=&{\Omega}_{n}(x_{n})-K\frac{\eta_{0}}{1+\eta_{0}}\lambda_{n}\beta_{n} g(x_{n})+K\varphi_{n}\\ &=& f(x_{n})+h(x_{n})+\left( 1-K\frac{\eta_{0}}{1+\eta_{0}}\lambda_{n}\right)\beta_{n}g(x_{n})+K\varphi_{n} \quad \forall n\geq1. \end{array} $$

### **Lemma 3**

*Let* $u\in \mathcal {S}$. *According to the first-order optimality conditions, there exist* *v* ∈ *∂* *f*(*u*)*and* *p* ∈ *N*_arg min *g*_(*u*),*such that* 0 = *v* + ∇*h*(*u*) + *p*.*Then, for every* *n* ≥ 2, *it holds that*

15 $$\begin{array}{@{}rcl@{}} &&{\Gamma}_{n+1}-{\Gamma}_{n} - \alpha({\Gamma}_{n}-{\Gamma}_{n-1})+K\lambda_{n}\beta_{n}\left[\frac{2}{L_{g}(1+\eta_{0})}-(1+\eta_{0})\lambda_{n}\beta_{n}\right]\!\!\|\nabla g(x_{n})\|^{2}\\ &\leq&\left[\frac{\alpha}{2\lambda_{n}}-\frac{1}{\lambda_{n}}+\frac{L_{h}+\beta_{n} L_{g}}{2}+K\right]\|x_{n+1}-x_{n}\|^{2}\\ &&+\,\left[\frac{\alpha}{2\lambda_{n}}+2\alpha K\right]\|x_{n}-x_{n-1}\|^{2}+\frac{4K(1+\eta_{0})}{\eta_{0}}{\lambda_{n}^{2}}\|\nabla h(u)+v\|^{2}\\ &&+\,K\left[\frac{4(1+\eta_{0})}{\eta_{0}}{\lambda_{n}^{2}}-\frac{2\lambda_{n}}{L_{h}}\right]\|\nabla h(x_{n})-\nabla h(u)\|^{2}\\ &&+\,\frac{K\eta_{0}}{1+\eta_{0}}\lambda_{n}\beta_{n}\left[g^{*}\left( \frac{2p}{\frac{\eta_{0}}{1+\eta_{0}}\beta_{n}}\right)-\sigma_{\arg\min g}\left( \frac{2p}{\frac{\eta_{0}}{1+\eta_{0}}\beta_{n}}\right)\right]\\ &&+\,\alpha({\Omega}_{n-1}(x_{n-1})-{\Omega}_{n}(x_{n}))\,+\,\frac{\alpha K\eta_{0}}{1+\eta_{0}}(\lambda_{n}\beta_{n} g(x_{n})-\!\lambda_{n-1}\beta_{n-1}g(x_{n-1})).\\ \end{array} $$

### *Proof*

We write (2) for *η* := *η*_0_, multiply it by K, and after combining the resulting inequality with (13), we obtain for every *n* ≥ 1 that

$$\begin{array}{@{}rcl@{}} && {\Omega}_{n+1}(x_{n+1}) +K\varphi_{n+1}-{\Omega}_{n}(x_{n})-K\varphi_{n}+\frac{K\eta_{0}}{1+\eta_{0}}\lambda_{n}\beta_{n}g(x_{n})\\ && -\,\alpha(K\varphi_{n}-K\varphi_{n-1})+K\lambda_{n}\beta_{n}\left[\frac{2}{L_{g}(1+\eta_{0})}-(1+\eta_{0})\lambda_{n}\beta_{n}\right]\|\nabla g(x_{n})\|^{2}\\ &\leq & \left[\frac{\alpha}{2\lambda_{n}}-\frac{1}{\lambda_{n}}+\frac{L_{h}+\beta_{n} L_{g}}{2}+K\right]\|x_{n+1}-x_{n}\|^{2}+\left[\frac{\alpha}{2\lambda_{n}}+2\alpha K\right]\|x_{n}-x_{n-1}\|^{2}\\ &&+\,\frac{4K(1+\eta_{0})}{\eta_{0}}{\lambda_{n}^{2}}\|\nabla h(u)+v\|^{2}+K\left[\frac{4(1+\eta_{0})}{\eta_{0}}{\lambda_{n}^{2}}-\frac{2\lambda_{n}}{L_{h}}\right]\|\nabla h(x_{n})-\nabla h(u)\|^{2}\\ &&+\,(\beta_{n+1}-\beta_{n})g(x_{n+1})+\frac{K\eta_{0}}{1+\eta_{0}}\lambda_{n}\beta_{n}\left[g^{\ast}\left( \frac{2p}{\frac{\eta_{0}}{1+\eta_{0}}\beta_{n}}\right)-\sigma_{\arg\min g}\left( \frac{2p}{\frac{\eta_{0}}{1+\eta_{0}}\beta_{n}}\right)\right]. \end{array} $$

In view of Assumption 1(III), we deduce that

$$\begin{array}{@{}rcl@{}} && {\Omega}_{n+1}(x_{n+1}) +K\varphi_{n+1}-{\Omega}_{n}(x_{n})-K\varphi_{n}+\frac{K\eta_{0}}{1+\eta_{0}}\lambda_{n}\beta_{n}g(x_{n})\\ && -\,\alpha(K\varphi_{n}-K\varphi_{n-1})+K\lambda_{n}\beta_{n}\left[\frac{2}{L_{g}(1+\eta_{0})}-(1+\eta_{0})\lambda_{n}\beta_{n}\right]\|\nabla g(x_{n})\|^{2}\\ &\leq & \left[\frac{\alpha}{2\lambda_{n}}-\frac{1}{\lambda_{n}}+\frac{L_{h}+\beta_{n} L_{g}}{2}+K\right]\|x_{n+1}-x_{n}\|^{2}+\left[\frac{\alpha}{2\lambda_{n}}+2\alpha K\right]\|x_{n}-x_{n-1}\|^{2}\\ && +\,\frac{4K(1+\eta_{0})}{\eta_{0}}{\lambda_{n}^{2}}\|\nabla h(u)+v\|^{2}+K\left[\frac{4(1+\eta_{0})}{\eta_{0}}{\lambda_{n}^{2}}-\frac{2\lambda_{n}}{L_{h}}\right]\|\nabla h(x_{n})-\nabla h(u)\|^{2}\\ && +\,\frac{K\eta_{0}}{1+\eta_{0}}\lambda_{n+1}\beta_{n+1}g(x_{n+1})\\ && +\,\frac{K\eta_{0}}{1+\eta_{0}}\lambda_{n}\beta_{n}\left[g^{\ast}\left( \frac{2p}{\frac{\eta_{0}}{1+\eta_{0}}\beta_{n}}\right)-\sigma_{\arg\min g}\left( \frac{2p}{\frac{\eta_{0}}{1+\eta_{0}}\beta_{n}}\right)\right] \quad \forall n \geq 1 \end{array} $$

and further

$$\begin{array}{@{}rcl@{}} &&{\Gamma}_{n+1}-{\Gamma}_{n}-\alpha(K\varphi_{n}-K\varphi_{n-1})\\ && +\,K\lambda_{n}\beta_{n}\left[\frac{2}{L_{g}(1+\eta_{0})}-(1+\eta_{0})\lambda_{n}\beta_{n}\right]\|\nabla g(x_{n})\|^{2}\\ &\leq& \left[\frac{\alpha}{2\lambda_{n}}-\!\frac{1}{\lambda_{n}}+\!\frac{L_{h}+\beta_{n} L_{g}}{2}+K\right]\|x_{n+1}-x_{n}\|^{2}+\left[\frac{\alpha}{2\lambda_{n}}+2\alpha K\right]\|x_{n}-x_{n-1}\|^{2}\\ &&+\,\frac{4K(1+\eta_{0})}{\eta_{0}}{\lambda_{n}^{2}}\|\nabla h(u)+v\|^{2}+K\left[\frac{4(1+\eta_{0})}{\eta_{0}}{\lambda_{n}^{2}}-\frac{2\lambda_{n}}{L_{h}}\right]\|\nabla h(x_{n})-\nabla h(u)\|^{2}\\ &&+\,\frac{K\eta_{0}}{1+\eta_{0}}\lambda_{n}\beta_{n}\left[g^{\ast}\left( \frac{2p}{\frac{\eta_{0}}{1+\eta_{0}}\beta_{n}}\right)-\sigma_{\arg\min g}\left( \frac{2p}{\frac{\eta_{0}}{1+\eta_{0}}\beta_{n}}\right)\right] \quad \forall n\geq2. \end{array} $$

In order to obtain (15), we only have to add *α*(Ω_*n*−1_(*x*_*n*−1_) −Ω_*n*_(*x*_*n*_)) and $\frac {\alpha K\eta _{0}}{1+\eta _{0}}(\lambda _{n}\beta _{n}g(x_{n})-\lambda _{n-1}\beta _{n-1}g(x_{n-1}))$ to both sides of the above inequality. □

The following results is a very useful tool in the convergence analysis of inertial algorithms (see [1, 2, 20]).

### **Lemma 4**

*Let* $\{a_{n}\}_{n=0}^{\infty }$ , $\{b_{n}\}_{n=1}^{\infty }$ *and* $\{c_{n}\}_{n=1}^{\infty }$ *be real sequences* *and* *α* ∈ [0, 1)*be a given real* *number. Assume that* $\{a_{n}\}_{n=1}^{\infty }$ *is bounded from below,* $\{b_{n}\}_{n=1}^{\infty }$ *is nonnegative and* ${\sum }_{n=1}^{\infty } c_{n}<+\infty ,$ *such* *that*

$$a_{n+1}-a_{n}-\alpha(a_{n}-a_{n-1})+b_{n}\leq c_{n} \quad \forall n \geq 1. $$

*Then the following statements hold:*

(i) ${\sum }_{n=1}^{\infty }[a_{n}-a_{n-1}]_{+}<+\infty $, *where* [*t*]_+_ := max{*t*,0};
(ii) $\{a_{n}\}_{n=1}^{\infty }$ *converges and* ${\sum }_{n=1}^{\infty } b_{n}<+\infty $.

The results presented in Lemma 5 related to the convergence of the generated iterates and in Lemma 6 related to the convergence of the objective function values will be used in the next section in the proof of the main theorem in combination with the Opial lemma stated in Lemma 7.

### **Lemma 5**

*Let* $u\in \mathcal {S}$. *According to the first-order optimality conditions there exist* *v* ∈ *∂* *f*(*u*)*and* *p* ∈ *N*_arg min *g*_(*u*)*, such* *that* 0 = *v* + ∇*h*(*u*) + *p*.*Then the following statements are true:*

(i) *The sequence* $\{{\Gamma }_{n}\}_{n=1}^{+\infty }$ *is bounded from below;*
(ii) ${\sum }_{n=1}^{\infty }\|x_{n+1}-x_{n}\|^{2}<+\infty $ *;*
(iii) $\lim _{n\to +\infty }{\Gamma }_{n}$ *exists and* ${\sum }_{n=1}^{+\infty }\lambda _{n}\beta _{n}\|\nabla g(x_{n})\|^{2}<+\infty $ *;*
(iv) $\lim _{n\to +\infty }\|x_{n}-u\|$ *exists,* ${\sum }_{n=1}^{\infty }[\varphi _{n}-\varphi _{n-1}]_{+}\!<+\infty $ *and* ${\sum }_{n=1}^{\infty }\lambda _{n}\beta _{n}g(x_{n})\!<+\infty $ *;*
(v) $\lim _{n\to +\infty }{\Omega }_{n}(x_{n})$ *exists;*
(vi) $\lim _{n\to +\infty }g(x_{n})=0$ *and every sequential weak cluster point of* $\{x_{n}\}_{n=1}^{\infty }$ *lies in* arg min*g*.

### *Proof*

(i) According to Assumption 1(III) we have that

$$\frac{L_{h}+\beta_{n}L_{g}}{2}+\frac{\alpha-1}{\lambda_{n}}\leq -(1+2\alpha)K-c, $$

which implies that 1 ≥ *K* *λ*_*n*_ and further

16 $$ \left( 1- \frac{K\eta_{0}}{1+\eta_{0}}\lambda_{n}\right)\beta_{n}g(x_{n})\geq0 \quad \forall n\geq1. $$

By using the definition of Γ_*n*_ and Assumption 1(II), we easily derive that $\{{\Gamma }_{n}\}_{n=1}^{\infty }$ is bounded from below.

(ii) For every *n* ≥ 2, we set

$$\mu_{n}:={\Gamma}_{n}-\alpha{\Gamma}_{n-1}+\left( \frac{1}{2\lambda_{n}}+2K\right)\alpha\|x_{n}-x_{n-1}\|^{2} $$

and

$$\omega_{n}:=\alpha({\Omega}_{n-1}(x_{n-1})-{\Omega}_{n}(x_{n}))+\frac{\alpha K\eta_{0}}{1+\eta_{0}}(\lambda_{n}\beta_{n} g(x_{n})-\lambda_{n-1}\beta_{n-1}g(x_{n-1})). $$

For a fixed natural number *N*_0_ ≥ 2, it holds that

$$\begin{array}{@{}rcl@{}} \frac{1}{\alpha}\sum\limits_{n=2}^{N_{0}}\omega_{n}&=&f(x_{1})+h(x_{1})+\left( 1-\frac{K\eta_{0}}{1+\eta_{0}}\lambda_{1}\right)\beta_{1}g(x_{1})-f(x_{N_{0}})-h(x_{N_{0}})\\ &&-\left( 1-\frac{K\eta_{0}}{1+\eta_{0}}\lambda_{N_{0}}\right)\beta_{N_{0}}g(x_{N_{0}}). \end{array} $$

Since *f* + *h* is bounded from below and relation (16) is true, we obtain that $\{\omega _{n}\}_{n=1}^{\infty }$ is summable.

For every *n* ≥ 1, we set

$$\delta_{n}:=\left( \frac{1}{2\lambda_{n}}+2K\right)\alpha+c. $$

Consequently, according Assumption 1(III), it follows that

17 $$ \frac{L_{h}+\beta_{n} L_{g}}{2}+\frac{\alpha}{2\lambda_{n}}-\frac{1}{\lambda_{n}}+K\leq -\delta_{n} \quad \forall n\geq1. $$

Further, for every *n* ≥ 1, it holds

$$-\delta_{n}+\alpha\left( \frac{1}{2\lambda_{n+1}}+2K\right) = \frac{\alpha}{2}\left( \frac{1}{\lambda_{n+1}}-\frac{1}{\lambda_{n}}\right)-c, $$

which, together with Assumption 1(IV), implies that

18 $$ -\delta_{n}+\alpha\left( \frac{1}{2\lambda_{n+1}}+2K\right)\leq 1-c $$

and so

19 $$ \delta_{n+1}\leq \delta_{n}+1. $$

On the other hand, by Assumption 1(III), we also have for every *n* ≥ 1

20 $$ 0<\frac{2}{L_{g}(1+\eta_{0})}-(1+\eta_{0})b\leq\frac{2}{L_{g}(1+\eta_{0})}-(1+\eta_{0})\lambda_{n}\beta_{n} $$

and so

$$K\lambda_{n}\beta_{n}\left[\frac{2}{L_{g}(1+\eta_{0})}-(1+\eta_{0})\lambda_{n}\beta_{n}\right]\geq0. $$

By employing the last inequality in Lemma 3 we obtain for every *n* ≥ 2

$$\begin{array}{@{}rcl@{}} \mu_{n+1}-\mu_{n} &=& {\Gamma}_{n+1}-{\Gamma}_{n}-\alpha\left( {\Gamma}_{n}-{\Gamma}_{n-1}\right)+\alpha\left( \frac{1}{2\lambda_{n+1}}+2K\right)\|x_{n+1}-x_{n}\|^{2}\\ && -\,\alpha\left( \frac{1}{2\lambda_{n}}+2K\right)\|x_{n}-x_{n-1}\|^{2}\\ &\leq& \left[\frac{\alpha}{2\lambda_{n}}-\frac{1}{\lambda_{n}}+\frac{L_{h}+\beta_{n} L_{g}}{2}+K+\alpha\left( \frac{1}{2\lambda_{n+1}}+2K\right)\right]\|x_{n+1}-x_{n}\|^{2}\\ &&+\,\frac{4K(1+\eta_{0})}{\eta_{0}}{\lambda_{n}^{2}}\|\nabla h(u)+v\|^{2}\\ &&+\,K\left[\frac{4(1+\eta_{0})}{\eta_{0}}{\lambda_{n}^{2}}-\frac{2\lambda_{n}}{L_{h}}\right]\|\nabla h(x_{n})-\nabla h(u)\|^{2}\\ &&+\,\frac{K\eta_{0}}{1+\eta_{0}}\lambda_{n}\beta_{n}\left[g^{\ast}\left( \frac{2p}{\frac{\eta_{0}}{1+\eta_{0}}\beta_{n}}\right)-\sigma_{\arg\min g}\left( \frac{2p}{\frac{\eta_{0}}{1+\eta_{0}}\beta_{n}}\right)\right]+\omega_{n}\\ &\leq& (1-c)\|x_{n+1}-x_{n}\|^{2}+\frac{4K(1+\eta_{0})}{\eta_{0}}{\lambda_{n}^{2}}\|\nabla h(u)+v\|^{2}\\ &&+\,K\left[\frac{4(1+\eta_{0})}{\eta_{0}}{\lambda_{n}^{2}}-\frac{2\lambda_{n}}{L_{h}}\right]\|\nabla h(x_{n})-\nabla h(u)\|^{2}\\ &&+\,\frac{K\eta_{0}}{1+\eta_{0}}\lambda_{n}\beta_{n}\left[g^{\ast}\left( \frac{2p}{\frac{\eta_{0}}{1+\eta_{0}}\beta_{n}}\right)-\sigma_{\arg\min g}\left( \frac{2p}{\frac{\eta_{0}}{1+\eta_{0}}\beta_{n}}\right)\right]+\omega_{n}, \end{array} $$

where for the last inequality we use (17) and (18). Since *λ*_*n*_ → 0 as *n* → + *∞*, there exists $N_{1}\in \mathbb {N}$, such that for every *n* ≥ *N*_1_, we have $\frac {4(1+\eta _{0})}{\eta _{0}}\lambda _{n}-\frac {2}{L_{h}}<0$. This implies that for every *n* ≥ *N*_1_

$$\begin{array}{@{}rcl@{}} \mu_{n+1}-\mu_{n} &\leq& (1-c)\|x_{n+1}-x_{n}\|^{2}+\frac{4K(1+\eta_{0})}{\eta_{0}}{\lambda_{n}^{2}}\|\nabla h(u)+v\|^{2}\\ &&+\,\frac{K\eta_{0}}{1+\eta_{0}}\lambda_{n}\beta_{n}\left[g^{\ast}\left( \frac{2p}{\frac{\eta_{0}}{1+\eta_{0}}\beta_{n}}\right) - \sigma_{\arg\min g}\left( \frac{2p}{\frac{\eta_{0}}{1+\eta_{0}}\beta_{n}}\right)\right]+\omega_{n}. \end{array} $$

Summing up this inequality for *n* = *N*_1_,…,*N*_2_, where *N*_2_ is a natural number with *N*_2_ ≥ *N*_1_, we obtain that

21 $$\begin{array}{@{}rcl@{}} \mu_{N_{2}+1}-\mu_{N_{1}} &\leq& (1-c)\sum\limits_{n=N_{1}}^{N_{2}}\|x_{n+1}-x_{n}\|^{2}+\frac{4K(1+\eta_{0})}{\eta_{0}}\|\nabla h(u)+v\|^{2}\sum\limits_{n=N_{1}}^{N_{2}}{\lambda_{n}^{2}}\\ &&+\,\frac{K\eta_{0}}{1+\eta_{0}}\sum\limits_{n=N_{1}}^{N_{2}}\lambda_{n}\beta_{n}\left[g^{\ast}\left( \frac{2p}{\frac{\eta_{0}}{1+\eta_{0}}\beta_{n}}\right)-\sigma_{\arg\min g}\left( \frac{2p}{\frac{\eta_{0}}{1+\eta_{0}}\beta_{n}}\right)\right]\\ &&+\,\sum\limits_{n=N_{1}}^{N_{2}}\omega_{n}. \end{array} $$

This means that $\{\mu _{n}\}_{n=2}^{\infty }$ is bounded from above (we take into account that *c* > 1). Let *M* be a positive upper bound of $\{\mu _{n}\}_{n=2}^{\infty }$. Observing that Γ_*n*+1_ − *α*Γ_*n*_ ≤ *μ*_*n*+1_ ≤ *M*, thus Γ_*n*+1_ ≤ *α*Γ_*n*_ + *M* for every *n* ≥ *N*_1_, we obtain

$${\Gamma}_{n}\leq\alpha^{n-N_{1}}{\Gamma}_{N_{1}}+M\sum\limits_{k=1}^{n-N_{1}}\alpha^{k-1}\leq \alpha^{n-N_{1}}{\Gamma}_{N_{1}}+\frac{M}{1-\alpha} \quad \forall n\geq N_{1}+1. $$

Since $\{{\Gamma }_{n}\}_{n=1}^{\infty }$ is bounded from below, there exists $C\in \mathbb {R}$, such that

$$\begin{array}{@{}rcl@{}} -\mu_{N_{2}+1}&=&-{\Gamma}_{N_{2}+1}+\alpha{\Gamma}_{N_{2}}-\left( \frac{1}{2\lambda_{N_{2}+1}}+2K\right)\alpha\|x_{N_{2}+1}-x_{N_{2}}\|^{2}\\ &\leq& \alpha{\Gamma}_{N_{2}}+C \leq \alpha^{N_{2}-N_{1}+1}{\Gamma}_{N_{1}}+\frac{M\alpha}{1-\alpha}+C \quad \forall N_{2} \geq N_{1}. \end{array} $$

Thus, from the inequality (21), by taking into account that *c* > 1, we deduce that

$$\sum\limits_{n=1}^{+\infty}\|x_{n+1}-x_{n}\|^{2}<+\infty. $$

(iii) From Lemma 3, by using (17), (19), and (20), we obtain

$$\begin{array}{@{}rcl@{}} &&{\Gamma}_{n+1}-{\Gamma}_{n} - \alpha({\Gamma}_{n}-{\Gamma}_{n-1})+K\lambda_{n}\beta_{n}\left[\frac{2}{L_{g}(1+\eta_{0})}-(1+\eta_{0})b\right]\|\nabla g(x_{n})\|^{2}\\ &\leq&-\delta_{n}\|x_{n+1}-x_{n}\|^{2}+\delta_{n-1}\|x_{n}-x_{n-1}\|^{2}+\frac{4K(1+\eta_{0})}{\eta_{0}}{\lambda_{n}^{2}}\|\nabla h(u)+v\|^{2}\\ &&+\,\frac{K\eta_{0}}{1+\eta_{0}}\lambda_{n}\beta_{n}\left[g^{\ast}\left( \frac{2p}{\frac{\eta_{0}}{1+\eta_{0}}\beta_{n}}\right)-\sigma_{\arg\min g}\left( \frac{2p}{\frac{\eta_{0}}{1+\eta_{0}}\beta_{n}}\right)\right]+\omega_{n} \quad \forall n \geq N_{1}. \end{array} $$

Since $\{{\Gamma }_{n}\}_{n=1}^{\infty }$ is bounded from below, by using Lemma 4, it follows that (iii) is true.

(iv) The statement follows from Lemma 1 and Lemma 4.

(v) Thanks to (iii), (iv) and ${\Gamma }_{n}={\Omega }_{n}(x_{n})-K\frac {\eta _{0}}{1+\eta _{0}}\lambda _{n}\beta _{n} g(x_{n})+K\varphi _{n}$ for every *n* ≥ 1, we obtain that $\lim _{n\to +\infty }{\Omega }_{n}(x_{n})$ exists.

(vi) Since *λ*_*n*_ *β*_*n*_ ≥ *a* > 0 for every *n* ≥ 1, we have ${\sum }_{n=1}^{+\infty }g(x_{n})<+\infty $ and so $\lim _{n\to +\infty }g(x_{n})=0$.

Finally, let $y\in {\mathcal H}$ be a sequential weak cluster point of $\{x_{n}\}_{n=1}^{\infty }$ and $\{x_{n_{j}}\}_{j=1}^{\infty }$ be a subsequence of $\{x_{n}\}_{n=1}^{\infty }$, such that $x_{n_{j}}$ weakly converges to *y* as *j* → + *∞*. Since *g* is weakly lower semicontinuous, we obtain that

$$g(y)\leq\liminf_{j\to+\infty}g(x_{n_{j}})\leq \lim_{n\to+\infty}g(x_{n})=0, $$

which means that *y* ∈ arg min *g*. □

### **Lemma 6**

*Let* $u\in \mathcal {S}$ *. Then, we* *have*

$$-\infty \leq \sum\limits_{n=1}^{\infty}\lambda_{n}\left[{\Omega}_{n+1}(x_{n+1})-(f(u)+h(u))\right]<+\infty. $$

### *Proof*

For every *n* ≥ 1, we have that

$$\begin{array}{@{}rcl@{}} {\Omega}_{n+1}(x_{n+1})-(f(u)+h(u)) &=&f(x_{n+1})+h(x_{n})+\beta_{n}g(x_{n})-(f(u)+h(u))\\ &&+\,(h(x_{n+1})-h(x_{n}))+(\beta_{n+1}-\beta_{n})g(x_{n+1})\\ &&+\,\beta_{n}(g(x_{n+1})-g(x_{n})) \\ &\leq&f(x_{n+1})+h(x_{n})+\beta_{n}g(x_{n})-(f(u)+h(u))\\ &&+\,(h(x_{n+1})-h(x_{n}))+\frac{K\eta_{0}}{1+\eta_{0}}\lambda_{n+1}\beta_{n+1}g(x_{n+1})\\ &&+\,\beta_{n}(g(x_{n+1})-g(x_{n})) \end{array} $$

and so

$$\begin{array}{@{}rcl@{}} \lambda_{n}[{\Omega}_{n+1}(x_{n+1})-(f(u)+h(u))] &\leq&\lambda_{n}[f(x_{n+1})+h(x_{n})+\beta_{n}g(x_{n})-(f(u)+h(u))]\\ &&+\,\lambda_{n}[(h(x_{n+1})-h(x_{n})]\\&&+\,\frac{K\eta_{0}}{1+\eta_{0}}\lambda_{n}\lambda_{n+1}\beta_{n+1}g(x_{n+1})\\ &&+\,\lambda_{n}\beta_{n}[g(x_{n+1})-g(x_{n})]. \end{array} $$

According to the Descent Lemma we have for every *n* ≥ 1

$$\begin{array}{@{}rcl@{}} \lambda_{n}[h(x_{n+1})-h(x_{n})] &\leq&\lambda_{n}\langle \nabla h(x_{n}), x_{n+1}-x_{n}\rangle+\frac{\lambda_{n} L_{h}}{2}\|x_{n+1}-x_{n}\|^{2}\\ &\leq&\frac{{\lambda_{n}^{2}}}{2}\|\nabla h(x_{n})\|^{2}+\frac{1+\lambda_{n} L_{h}}{2}\|x_{n+1}-x_{n}\|^{2} \end{array} $$

and

$$\begin{array}{@{}rcl@{}} \lambda_{n}\beta_{n}[g(x_{n+1})-g(x_{n})]&\leq&\lambda_{n}\beta_{n}\langle \nabla g(x_{n}), x_{n+1}-x_{n}\rangle+\frac{\lambda_{n}\beta_{n} L_{g}}{2}\|x_{n+1}-x_{n}\|^{2}\\ &\leq&\frac{{\lambda_{n}^{2}}{\beta_{n}^{2}}}{2}\|\nabla g(x_{n})\|^{2}+\frac{1+\lambda_{n}\beta_{n} L_{g}}{2}\|x_{n+1}-x_{n}\|^{2}, \end{array} $$

which give rise to the following estimate

22 $$\begin{array}{@{}rcl@{}} \lambda_{n}[{\Omega}_{n+1}(x_{n+1})-(f(u)+h(u))] &\leq&\lambda_{n}[f(x_{n+1})+h(x_{n})+\beta_{n}g(x_{n})-(f(u)+h(u))]\\ &&+\,\frac{{\lambda_{n}^{2}}}{2}\|\nabla h(x_{n})\|^{2}\\ &&+\,\frac{2+\lambda_{n} (L_{h}+\beta_{n} L_{g})}{2}\|x_{n+1}-x_{n}\|^{2}\\ &&+\,\frac{K\eta_{0}}{1+\eta_{0}}\lambda_{n}\lambda_{n+1}\beta_{n+1}g(x_{n+1})\\ &&+\,\frac{{\lambda_{n}^{2}}{\beta_{n}^{2}}}{2}\|\nabla g(x_{n})\|^{2} \quad \forall n\geq1. \end{array} $$

Further, we notice that for every *n* ≥ 1

$$f(x_{n+1})-f(u)\leq \left\langle \frac{y_{n}-x_{n+1}}{\lambda_{n}}-\nabla h(x_{n})-\beta_{n}\nabla g(x_{n}),x_{n+1}-u\right\rangle $$

or, equivalently,

23 $$\begin{array}{@{}rcl@{}} &&\left\langle \frac{y_{n}-x_{n+1}}{\lambda_{n}},u-x_{n+1}\right\rangle - \langle \nabla h(x_{n}),u-x_{n+1}\rangle-\beta_{n}\langle \nabla g(x_{n}),u-x_{n+1}\rangle \\ &&\qquad \leq f(u)-f(x_{n+1}). \end{array} $$

On the other hand, since *g*(*u*) = 0, we have for every *n* ≥ 1

$$0=g(u)\geq g(x_{n})+\langle \nabla g(x_{n}),u-x_{n}\rangle $$

or, equivalently,

24 $$ -\beta_{n}\langle \nabla g(x_{n}),u-x_{n+1}\rangle\geq \beta_{n}g(x_{n})+\beta_{n}\langle \nabla g(x_{n}),x_{n+1}-x_{n}\rangle. $$

Similarly, we have for every *n* ≥ 1

$$h(u)\geq h(x_{n})+\langle \nabla h(x_{n}),u-x_{n}\rangle, $$

which implies that

25 $$ -\langle \nabla h(x_{n}),u-x_{n+1}\rangle\geq h(x_{n})-h(u)+\langle \nabla h(x_{n}),x_{n+1}-x_{n}\rangle. $$

By summing up the inequalities in (23)–(25), we obtain for every *n* ≥ 1

26 $$\begin{array}{@{}rcl@{}} 2\lambda_{n}[f(x_{n+1})+h(x_{n})+\beta_{n}g(x_{n})&-&(f(u)+h(u))] \leq 2\langle y_{n}-x_{n+1},x_{n+1}-u\rangle\\ &&+\,2\lambda_{n}\beta_{n}\langle \nabla g(x_{n}),x_{n}-x_{n+1}\rangle\\ &&+\,2\lambda_{n}\langle \nabla h(x_{n}),x_{n}-x_{n+1}\rangle\\ &\leq&2\langle y_{n}-x_{n+1},x_{n+1}-u\rangle\\ &&+\,{\lambda_{n}^{2}}{\beta_{n}^{2}}\|\nabla g(x_{n})\|^{2}+2\|x_{n}-x_{n+1}\|^{2}\\ &&+\,{\lambda_{n}^{2}}\|\nabla h(x_{n})\|^{2}. \end{array} $$

Not least, according to (4) and (5), we obtain for every *n* ≥ 1

$$\begin{array}{@{}rcl@{}} 2\langle y_{n}-x_{n+1},x_{n+1}-u\rangle &=&2\langle x_{n}-x_{n+1},x_{n+1}-u\rangle+2\alpha\langle x_{n}-x_{n-1},x_{n+1}-u\rangle\\ &\leq&\varphi_{n}-\varphi_{n+1}+\alpha(\varphi_{n}-\varphi_{n-1})\\ &&+\,2\alpha\|x_{n}-x_{n-1}\|^{2}+(\alpha-1)\|x_{n+1}-x_{n}\|^{2}\\ &\leq &\varphi_{n}-\varphi_{n+1}+\alpha(\varphi_{n}-\varphi_{n-1})+2\alpha\|x_{n}-x_{n-1}\|^{2}, \end{array} $$

which, combined with (26) and Lemma 5(iv), implies that

$$\sum\limits_{n=1}^{\infty}\lambda_{n}[f(x_{n+1})+h(x_{n})+\beta_{n}g(x_{n})-(f(u)+h(u))]<+\infty. $$

The conclusion follows by taking into account (22). □

## Convergence of the Iterates and of the Objective Function Values

In this section, we will prove the main result of this paper. This addresses the convergence of both the sequence of iterates $\{x_{n}\}_{n=1}^{\infty }$ generated by Algorithm 1 and of the sequence of objective values $\{f(x_{n})+h(x_{n})\}_{n=1}^{\infty }$.

The Opial lemma, which we state next and for which we refer to [12, Lemma 2.39], will play a crucial role in the convergence analysis.

### **Lemma 7**

*Let* ${\mathcal H}$ *be a real* *Hilbert space,* $C\subseteq {\mathcal H}$ *a* *nonempty set and* $\{x_{n}\}_{n=1}^{\infty }$ *a given sequence, such that:*

(i) *For every* *z* ∈ *C*, $\lim _{n\to +\infty }\|x_{n}-z\|$ *exists.*
(ii) *Every sequential weak cluster point of* $\{x_{n}\}_{n=1}^{\infty }$ *lies in* C.

*Then, the sequence* $\{x_{n}\}_{n=1}^{\infty }$ *converges weakly to a point in* C.

### **Theorem 1**

*Let* $\{x_{n}\}_{n=1}^{\infty }$ *be the sequence generated by Algorithm 1. Then:*

(i) *the sequence* $\{x_{n}\}_{n=1}^{\infty }$ *converges weakly to a point in* $\mathcal {S}$ *;*
(ii) *the sequence* $\{f(x_{n})+h(x_{n})\}_{n=1}^{\infty }$ *converges to the optimal objective value of the optimization problem*(1).

### *Proof*

(i) We know that $\lim _{n\to +\infty }\|x_{n}-u\|$ exists for all $u\in \mathcal {S}$ (see Lemma 5(iv)); hence, in view of the Opial lemma, it is sufficient to show that all sequential weak cluster points of $\{x_{n}\}_{n=1}^{\infty }$ are in $\mathcal {S}$. Since $\{\lambda _{n}\}_{n=1}^{\infty }\notin \ell ^{1}$ and $\lim _{n\to +\infty }{\Omega }_{n}(x_{n})$ exists, from Lemma 6, we obtain that

$$\lim_{n\to+\infty}{\Omega}_{n}(x_{n})\leq f(u)+h(u) \quad \forall u\in\mathcal{S}. $$

Let $x^{\ast }\in {\mathcal H}$ be a sequential weak cluster point of $\{x_{n}\}_{n=1}^{\infty }$ and $\{x_{n_{k}}\}_{k=1}^{\infty }$ be a subsequence of $\{x_{n}\}_{n=1}^{\infty }$, such that $x_{n_{k}}$ converges weakly to *x* ^∗^ as *k* → + *∞*. From here, by Lemma 5(vi), we obtain that *x* ^∗^∈ arg min *g*. Take an arbitrary $u\in \mathcal {S}$. The weak lower semicontinuity of *f* and *h* implies that

$$\begin{array}{@{}rcl@{}} f(x^{\ast})+h(x^{\ast}) &\leq&\liminf_{k\to+\infty}f(x_{n_{k}})+\liminf_{k\to+\infty}h(x_{n_{k}})\\ &\leq&\liminf_{k\to+\infty}\left[f(x_{n_{k}})+h(x_{n_{k}})\right]\\ &\leq&\lim_{n\to+\infty}{\Omega}_{n}(x_{n})\leq f(u)+h(u)\\ & = &\min\{f(x)+h(x):x\in\arg\min g\} \end{array} $$

which means that $x^{\ast }\in \mathcal {S}$.

(ii) The statement is a direct consequence of the above inequalities. □

We close the paper by a remark which discusses the fulfillment of the conditions stated in Assumption 1.

### *Remark 2*

We chose

$$\eta_{0}\in (0,+\infty),\quad c\in(1,+\infty),\quad q\in \left( \frac{1}{2},1\right),\quad \alpha\in\left( 0, 1-\frac{1}{2(1+\eta_{0})^{2}}\right) $$

and

$$\gamma\in\left( \frac{1}{L_{g}(1-\alpha)(1+\eta_{0})^{2}},\min\left\{\frac{2}{L_{g}},\frac{3}{L_{g}(1-\alpha)(1+\eta_{0})^{2}}\right\}\right). $$

We set

$$K:=\frac{2(1+\eta_{0})}{\alpha\eta_{0}}, $$

$$\beta_{n}:=\frac{\gamma\left[L_{h}+2((1+2\alpha)K+c)\right]}{2-\gamma L_{g}}+\left[\frac{(1-\alpha)\gamma L_{g}(1+\eta_{0})^{2}-1}{L_{g}(1+\eta_{0})^{2}}\right]\frac{K\eta_{0}}{1+\eta_{0}}n^{q}, $$

and

$$\lambda_{n}:=\frac{(1-\alpha)\gamma}{\beta_{n}}-\frac{1}{\beta_{n} L_{g} (1+\eta_{0})^{2}} $$

for every *n* ≥ 1.

(i) By taking $a:=(1-\alpha )\gamma -\frac {1}{L_{g}(1+\eta _{0})^{2}}$ and *b* > 0, such that $a<b<\frac {2}{L_{g}(1+\eta _{0})^{2}}$, we have
  $$0<a=\lambda_{n}\beta_{n}< b<\frac{2}{L_{g}(1+\eta_{0})^{2}} \quad \forall n\geq1. $$
(ii) Since $\beta _{n}\geq \frac {\gamma [L_{h}+2((1+2\alpha )K+c)]}{2-\gamma L_{g}}$, we have $\frac {L_{h}+\beta _{n} L_{g}}{2}-\frac {\beta _{n}}{\gamma }\leq -(1+2\alpha )K-c$ for every *n* ≥ 1. On the other hand, since $\frac {\beta _{n}}{\gamma }\leq \frac {(1-\alpha )}{\lambda _{n}}$, we have that $\frac {L_{h}+\beta _{n}L_{g}}{2}+\frac {\alpha -1}{\lambda _{n}}\leq -(1+2\alpha )K-c$ for every *n* ≥ 1.
(iii) For every *n* ≥ 1, we also have
  $$\begin{array}{@{}rcl@{}} \beta_{n+1}-\beta_{n} &=&\left[\frac{(1-\alpha)\gamma L_{g}(1+\eta_{0})^{2}-1}{L_{g}(1+\eta_{0})^{2}}\right]\frac{K\eta_{0}}{1+\eta_{0}}\left( (n+1)^{q}-n^{q}\right)\\ &\leq&\left[\frac{(1-\alpha)\gamma L_{g}(1+\eta_{0})^{2}-1}{L_{g}(1+\eta_{0})^{2}}\right]\frac{K\eta_{0}}{1+\eta_{0}}\\ &=&\frac{K\eta_{0}}{1+\eta_{0}}\lambda_{n+1}\beta_{n+1}. \end{array} $$
(iv) From (iii), it follows that for every *n* ≥ 1
  $$\frac{1}{\lambda_{n+1}}-\frac{1}{\lambda_{n}} = \left( \beta_{n+1}-\beta_{n}\right)\left[\frac{L_{g}(1+\eta_{0})^{2}}{(1-\alpha)\gamma L_{g}(1+\eta_{0})^{2}-1}\right]\leq\frac{K\eta_{0}}{1+\eta_{0}}=\frac{2}{\alpha}. $$
(v) Due to the fact that $q\in \left (\frac {1}{2},1\right )$, we have ${\sum }_{n=1}^{+\infty }\frac {1}{\beta _{n}}=+\infty $ and ${\sum }_{n=1}^{+\infty }\frac {1}{{\beta _{n}^{2}}}<+\infty $. Consequently, $\{\lambda _{n}\}_{n=1}^{\infty }\in \ell ^{2}\setminus \ell ^{1}$.
(vi) Since *g* ≤ *δ*_arg min *g*_, it holds that *g* ^∗^≥ (*δ*_arg min *g*_)^∗^ = *σ*_arg min *g*_ and so *g* ^∗^− *σ*_arg min *g*_ ≥ 0. For a function $g: {\mathcal H} \rightarrow \mathbb {R}$ fulfilling $g \geq \frac {a}{2}\text {dist}^{2}(\cdot ,\arg \min g)$ where *a* > 0, it holds that $g^{\ast }(x)-\sigma _{\arg \min g}(x)\leq \frac {1}{2a}\|x\|^{2}$ for every $x \in {\mathcal H}$. Thus, for every *n* ≥ 1,
  $$\lambda_{n}\beta_{n}\left[g^{\ast}\left( \frac{p}{\beta_{n}}\right)-\sigma_{\arg\min g}\left( \frac{p}{\beta_{n}}\right)\right]\leq \frac{\lambda_{n}}{2a\beta_{n}}\|p\|^{2} \quad \forall p\in\text{ran}(N_{\arg\min g}). $$
  Since ${\sum }_{n=1}^{\infty }\frac {1}{{\beta _{n}^{2}}}<+\infty $, from here it follows that
  $$\sum\limits_{n=1}^{\infty}\lambda_{n}\beta_{n}\left[g^{\ast}\left( \frac{p}{\beta_{n}}\right)-\sigma_{\arg\min g}\left( \frac{p}{\beta_{n}}\right)\right]<+\infty. $$

## Conclusions

We investigate the weak (non-ergodic) convergence of an inertial proximal-gradient method with penalization terms in connection with the solving of a bilevel optimization problem, having as objective the sum of a convex nonsmooth with a convex smooth function, and as constrained set the set of minimizers of another convex and differentiable function. The techniques of the proof combine tools specific to inertial algorithms [3] and penalty type methods [5, 8]. We show the convergence of both generated iterates and objective function values.
